# Dalbavancin as suppressive antibiotic therapy in patients with prosthetic infections: efficacy and safety

**DOI:** 10.3389/fphar.2023.1185602

**Published:** 2023-06-28

**Authors:** Andrés Ruiz-Sancho, María Núñez-Núñez, Laura Castelo-Corral, Francisco Javier Martínez-Marcos, Nagore Lois-Martínez, Mohd Hafiz Abdul-Aziz, David Vinuesa-García

**Affiliations:** ^1^ Infectious Diseases Department, University Hospital Clinico San Cecilio, Granada, Spain; ^2^ Biosanitary Research Institute, Ibs Granada, Granada, Spain; ^3^ Pharmacy Department, University Hospital Clinico San Cecilio, Granada, Spain; ^4^ Consortium for Biomedical Research in Epidemiology and Public Health (CIBERESP), Madrid, Spain; ^5^ Infectious Diseases Department, University Hospital Complexo A Coruña, A Coruña, Spain; ^6^ Infectious Diseases Department, Juan Ramón Jiménez Hospital, Huelva, Spain; ^7^ Infectious Diseases Department, Severo Ochoa Hospital, Madrid, Spain; ^8^ University of Queensland Centre for Clinical Research (UQCCR), Faculty of Medicine, The University of Queensland, Brisbane, QLD, Australia

**Keywords:** dalbavancin, Gram-positive prosthetic infection, suppressive antibiotic therapy, antimicrobial stewardship, antimicrobials

## Abstract

Suppressive antibiotic therapy (SAT) is a strategy to alleviate symptoms and/or to reduce the progression of an infection when other treatment options cannot be used. Dalbavancin, due to its prolonged half-life, enables (bi) weekly dosing. Here, we report our multicenter real-life clinical experience with dalbavancin used as SAT in patients with prosthetic joint or vascular infections. Medical records of all adult patients with documented vascular or orthopedic chronic prosthetic infections, who received dalbavancin as SAT between 2016 and 2018 from four Spanish hospitals were reviewed for inclusion. Descriptive analysis of demographic characteristics, Charlson Comorbidity index, Barthel index, isolated pathogens and indication, concomitant antibiotic use, adverse events, and clinical outcome of SAT were performed. Eight patients were eligible for inclusion, where six patients had prosthetic vascular infections (aortic valve) and two patients had knee prosthetic joint infections. The most common pathogens were *methicillin-susceptible Staphylococcus aureus* and *Enterococcus faecium*. All patients had a history of prior antibiotic treatment for the prosthetic infection [median duration of antibiotic days 125 days (IQR, 28–203 days)]. The median number of dalbavancin doses was 29 (IQR, 9–61) and concomitant antibiotic use (*n* = 5, 62.5%). Clinical success was reported in 75% (*n* = 6) of patients. Adverse events were reported in two patients (mild renal and hepatic impairment). The median estimated cost savings due to the avoided hospital days was €60185 (IQR, 19,916–94984) per patient. Despite the limitations of our study, this preliminary data provides valuable insight to support further evaluation of dalbavancin for SAT in patients with prosthetic infections in the outpatient setting when alternative treatments are not feasible.

## Introduction

In recent years, the aging population combined with improved surgical procedures had led to an increase in the use of prostheses and consequently, in an increase in the number of periprosthetic infections (Kurtz et al., 2007). Management of these infections often involves surgical intervention tailored to the clinical situation and prolonged high-dose antibiotics targeting the responsible microorganism(s) at the site of action and despite the efforts, available evidence regarding antimicrobial management remains low probably due to the high heterogeneity of clinical scenarios: location, presentation (acute or chronic), timing (early or delay after implantation), or microbiological isolates (empiric or targeted, mono/polymicrobial), among others (Ariza et al., 2017). The complexity of these infections requires a multidisciplinary approach to patient care, particularly when surgical clearance of the source of infection is not feasible. In such a scenario where the risk of infection relapse is high, prolonged suppressive antibiotic therapy (SAT) may be used to alleviate the symptoms and reduce the progression of the infection (Prendki et al., 2017; Roy and Grove, 2000). However, to our knowledge, there is no consensus on the antibiotic of choice, dose, method of administration, and duration of SAT (Segreti et al., 1998; Prendki et al., 2014; Lau et al., 2018).

Dalbavancin is one of the latest lipoglycopeptides approved for acute bacterial skin and skin structure infections (ABSSSI) by the U.S Food and Drug Administration (FDA) and the European Medicines Agency (EMA) (Xydalba, 2023). It is active against Gram-positive pathogens including *staphylococci*, *streptococci*, and *enterococci*—even methicillin-resistant isolates—and it has a unique pharmacokinetic (PK)/pharmacodynamic (PD) profile due to its highly protein-bound property (93%), particularly to albumin, leading to a steady-state volume of distribution of ≈ 10—12 L and a prolonged half-life ≈ 8.5 days. This allows for (bi) weekly administrations, and nowadays even longer periods are used, offering a convenient dosing schedule that may be advantageous in prolonged SAT when oral options are not available or contraindicated. Here, we aimed to share our multicenter, real-life clinical experience with intravenous dalbavancin used as SAT in patients with prosthetic joint or vascular infections.

## Methods

### Patient selection and study design

This was a multicenter retrospective cohort study conducted at four hospitals in Spain. The medical records of all adult patients (>18 years old) with documented vascular or orthopedic chronic prosthetic infections, who received dalbavancin as SAT between 2016 and 2018, were reviewed for inclusion.

### Data collection, variables, and definitions

Patient data were extracted from medical records by an infectious diseases physician at each participating hospital and all information was collected into a deidentified case report form. SAT was defined as the prescription of antibiotics for antimicrobial suppression.

Patient demographic characteristics (e.g., age, gender, body mass index [BMI], and renal function), Charlson Comorbidity Index, and the Barthel Index for Activities of Daily Living were collected. Relevant clinical and treatment-related variables including the site of infection, type of prosthesis used, and the isolated pathogens including microbiological susceptibility profile were recorded. The indication for SAT and the dalbavancin dosing regimen (dose, frequency, and duration), prior and concomitant antibiotic use, adverse events, and the clinical outcome of SAT were also collected.

Response to SAT and adverse events were recorded based on symptoms, temperature, inflammatory markers, potential drug-induced toxicity, and survival reported in medical records. Clinical outcome was defined as a “failure” if during the treatment with dalbavancin the patient presented recurrent signs of local infection (e.g., pain, swelling, erythema, fever, increased C-reactive protein, fistula, or purulent discharge), additional antibiotics were added, there was need of surgical interventions, or death due to the infection. Adverse events potentially related to dalbavancin were classified according to the Common Terminology Criteria for Adverse Events (CTCAE) (Cancer Institute and National, 2017). Potential reduction in length and cost of hospital stay was estimated using the duration of ambulatory administration of dalbavancin as reference. The average cost of an inpatient hospital day in Spain is €325/day and a 500 mg vial of dalbavancin is ∼€378 (Bouza et al., 2018).

### Statistical analysis

Discrete variables were expressed as counts (percentage) and continuous variables as medians and interquartile ranges (IQRs). Statistical analysis was performed using the statistical software package SPSS (V.18.0, IBM).

## Results

### Demographics and clinical data

Eight patients were included. Demographic, clinical, and treatment features of the patients are described in Table 1. The median (IQR) age and BMI were 73 years (56–80) and 24 (22–32), respectively. Most of the patients (5/8; 63%) were females. The indications for SAT in all patients were high surgical risk due to the patient’s age/comorbidities and the persistence of symptoms. Six patients had prosthetic vascular infections and the majority presented aortic valve involvement. Two patients had knee prosthetic joint infections, and in those, no concomitant bloodstream infections were reported, and both patients underwent surgical procedures including arthrotomy and synovectomy of the knee joint and 2-step replacement respectively.

**TABLE 1:** Clinical and treatment characteristics of patients on Dalbavancin as antibiotic suppressive therapy.

		Patients No							
		1	2	3	4	5	6	7	8
Sex		M	F	M	M	F	F	F	F
Age		78	47	80	75	85	53	65	71
BMI		24	22	23	38	22	33	28	21
Charlson index		9	3	4	3	7	2	6	9
Barthel index		100	100	95	65	65	100	100	30
Type of prosthesis		Vascular (aorta)	Vascular (cava)	Joint (knee)	Joint (Knee)	Vascular (aorta)	Vascular (aorta + valvular)	Vascular (aorta)	Vascular (aorta)
Indication for SAT		High surgical risk	High surgical risk	High surgical risk	High surgical risk	High surgical risk	High surgical risk	High surgical risk	High surgical risk
Type of treatment		Targeted	Targeted	Targeted	Targeted	Targeted	Targeted	Empirical	Targeted
Suspected causative pathogen		*E faecium*	*SAMS*	*S epidermidis*	*E faecium*	*S intermedius*	*S gallolyticus*	*Unknown*	*SAMS*
Bacteremia		Yes	Yes	No	No	Yes	Yes	Yes	Yes
Previous active ABT, days	DAP	5	No	23	135	0	0	94	25
	LZN	21	No	73	194	No	No	49	39
	Others	No	14	42	No	206	No	93	172
	Total duration	26	14	155	329	209	34	95	184
Dalbavancin (DAL)	Previous regimen outcome for DAL indication	Failure	Drug interactions	Failure + toxicity	No susceptible oral drugs	No susceptible oral drugs	Toxicity	Toxicity	Toxicity
	Initial dose, mg	1,000	1,000	1,000	1,000	1,500	1,500	1,500	1,500
	Maintenance dose, mg	500	500	500	500	1,500	1,500	1,500	1,500
	Frequency	7 days	7 days	7 days	7 days	14 days	14 days	14 days (5 doses)/21 days (36 doses)	14 days
	Total doses	62	13	48	87	7	16	42	7
Concomitant ABT		Minocycline	No	No	No	Rifampicin	No	Rifampicin	Rifampicin
Administration site		Day Hospital	Day Hospital	Day Hospital	Day Hospital	OPAT	Day Hospital	Day Hospital	Day Hospital
Adverse events (AE)		No	No	No	No	No	Asthenia	Liver and kidney injury	No
DAL interruption due to AE		No	No	No	No	No	No	Yes	No
Outcome		Successful	Successful	Successful	Failure—recurrence	Failure—exitus	Successful	Successful	Successful

*BMI: body mass index; AST: antimicrobial suppressive therapy; SAMS: *Staphylococcus aureus methicillin susceptible*; ABT: antibiotic; DAP: daptomycin; LNZ: linezolid; DAL: dalbavancin; OPAT: outpatient parenteral antimicrobial therapy.

### Infections and microorganisms

Seven patients (87.5%) received targeted dalbavancin treatment and the most common isolated pathogens were *methicillin-susceptible Staphylococcus aureus* and *Enterococcus faecium*. In most cases, initial isolates were susceptible to conventional antibiotics including daptomycin, glycopeptides, sulfamethoxazole/trimethoprim, and fluoroquinolones.

### Antibiotic therapy

All patients had a history of prior antibiotic treatment for the prosthetic infection. Daptomycin and linezolid were the most common antibiotics administered before dalbavancin SAT with a median duration of antibiotic days of 125 days (IQR, 28–203 days) prior to starting dalbavancin SAT.

The main reason for switching to dalbavancin in all cases was a more convenient parenteral posology. The cessation of prior antibiotics for four patients (50%) was due to changes in the susceptibility profile or failure of previous treatment and antibiotic resistance, in one patient (12.5%), this was due to drug-drug interactions (linezolid-serotonin reuptake inhibitors). In the remaining four patients (50%), the reason for switching the agent was a history of adverse events with previously administrated antimicrobials such as daptomycin-induced pulmonary toxicity, levofloxacin-induced tendinopathy and joint pain, and linezolid-induced anemia and liver injury.

Dalbavancin was administered as 1,000 mg intravenous (IV) on Day 1 followed by 500 mg IV once-weekly in four patients (50%). Three patients (37.5%) received 1,500 mg IV administered every 2 weeks and in one patient (12.5%), 1,500 mg IV was administered as an initial dose, followed by 1,500 mg IV bi-weekly for five doses and then 1,000 mg IV every 3 weeks until discontinuation. The median number of dalbavancin doses was 29 (IQR, 9–61 doses). The use of concomitant antibiotics with dalbavancin was present in five patients (62.5%) being rifampicin the most common antimicrobial used (3/5; 60%).

### Outcome and safety

Overall, six patients (75%) achieved clinical success with dalbavancin SAT. Treatment failure was observed in two patients (25%). The first patient who failed dalbavancin treatment underwent an initial two-step exchange procedure due to septic loosening of a knee prosthesis and persistent pain. *E. faecium* was persistently isolated in postoperative fistula cultures. The choice of continuing dalbavancin SAT was based on microbiology results (acquired resistance to linezolid during treatment course) and discharge options without an OPAT program. This patient received 18 months of dalbavancin SAT in total. A new two-step exchange procedure was conducted and 4 months more of dalbavancin were administered. Finally, supracondylar amputation of the lower limb was indicated due to recurrence and spacer fracture. The second patient who failed dalbavancin SAT was a poor candidate for explanting the endovascular device (abdominal aorta nellix endoprosthesis) because of the underlying severe medical problems and the high operative mortality risk. After multiple antibiotic regimens, the progression of infection was identified presenting an augmented size of periaortic collection plus a contiguity spondylodiscitis in L3-L4. Despite the fatal prognosis, dalbavancin was initiated as SAT as a palliative usage in an attempt to contain the endovascular descending aortic sealing endoprosthetic infection. *Streptococcus intermedius* was isolated in blood and periaortic and paravertebral collection cultures. The patient died 3 months after dalbavancin was initiated.

Adverse events were reported in two patients. One patient demonstrated mild asthenia, the other, progressive worsening of renal function. In this patient, serum creatinine increased to 2.1 mg/dL from the baseline value of 1.6 mg/dL, a difference of 0.5 mg/dL (Grade 1—CTCAE). This patient also demonstrated a mild increase in liver enzymes (GGT up to 199 UI; Grade 2—CTCAE) even when the concomitant drug (rifampin) was discontinued. Despite the mild severity of the adverse reaction, dalbavancin was discontinued.

### Cost

For our patients, we calculated that a total of 1,876 days of hospitalization could have been avoided by using dalbavancin instead of other parenteral drugs. The primary focus of this report was the clinical outcomes, and not to examine the cost of SAT with dalbavancin. Thus, to accurately evaluate its cost-effectiveness, more advanced and precise cost-effectiveness assessments will be required. Although a broad estimation by applying the criteria used in the cost analysis reported by Bouza et al. (2018) was made and excluding the patient on OPAT, considering a total of 282 doses and estimating a 7-day course per dose, our median of estimated cost savings was of €60185 (IQR, 19,916–94984) per patient due to the avoided hospital-days.

## Discussion

SAT is the therapy of choice when definitive surgical treatment options, including debridement, antibiotics, and implant retention (DAIR) seem to have failed or are dismissed (Pouderoux et al., 2019). SAT is generally very challenging for treating physicians because patients are frail with multiple comorbidities, have a short life expectancy, and are increasingly not so elderly (Prendki et al., 2017; Byren et al., 2009). The justification of SAT is not to eradicate the infection, but rather to alleviate symptoms and restore joint function by reducing the progression of the infection with implant retention (Zimmerli et al., 2004). It is a complex scenario with a lack of strong evidence and standardized definitions of outcomes (Cobo and del Pozo, 2011).

With this study, we aimed to share our experience in terms of safety and efficacy with dalbavancin used as long-term SAT in the outpatient setting for patients with Gram-positive prosthetic infections. After 8 years since dalbavancin was approved, its indication for only acute bacterial skin and skin structure infections remains. Although off-label usage for infections such as endocarditis, osteoarticular, and bloodstream infections is growing worldwide exponentially and thus studies, mostly observational studies, concerning its safety and efficacy are emerging. Data regarding adequate dalbavancin concentrations being achieved above the minimal inhibitory concentration (MIC) in blood and relevant tissues for most common Gram-positive pathogens support its use. Furthermore, there are little data describing resistance to dalbavancin thus far (Werth et al., 2018), and thanks to its prolonged half-life (∼8.5 days), weekly or biweekly administration allowed improved compliance and hospital length of stay (Núñez-Núñez et al., 2020).

The effectiveness of SAT is difficult to be established because of the difficulties in performing studies in this particular area of research. Randomized clinical trials are lacking and evidence to support long-term usage of dalbavancin is even more limited. Furthermore, the definition of endpoints across available studies is not standardized. Some investigators considered SAT to be successful if surgery is avoided while others required symptomatic relief (Segreti et al., 1998; Rao et al., 2003; Prendki et al., 2014). As a result of this, previous studies showed positive outcomes rates varying from 30% to 90% (Prendki et al., 2017). Considering that the goals of SAT are alleviation of the symptoms associated with the infection and slowing the progression of the infection, we took into account both criteria. We have included very different prosthetic infections with very different previous antimicrobial exposures. It would be necessary to segregate data according to indication and patient characteristics, and also to increase the sample size to verify real success rates; in our preliminary study, 75% of the patients had a positive/successful outcome. Leijtens et al. (2019), assessed retrospectively the clinical outcomes of 23 patients with a prosthetic joint infection after hip replacement treated with oral SAT started between 2006 and 2013 in a single center in Netherlands. Treatment was considered successful if no reoperation or death related to the infection occurred during the follow-up. After 33 months of treatment, in 13 patients (56.5%), SAT was considered successful, although wide differences among isolates were found and non-*S. aureus* infections success rate was 66% while lower if *S. aureus* was involved. In this study, 8.7% of patients had to end SAT due to side effects (Leijtens et al., 2019). Wouthuyzen-Bakker et al. (2017) in another Dutch center evaluated the efficacy of SAT in 21 patients with prosthetic joint infections. For this retrospective study, hip, knee, and shoulder prostheses were included and they observed a similar overall success rate of 67%. Although outcomes varied substantially from 90% to 50% according to patients with “standard” prosthesis (*n* = 11) or “tumor” prosthesis (*n* = 10) respectively. Authors considered treatment as failed “*when the patient still experienced joint pain, when surgical intervention (debridement, removal, arthrodesis, or amputation) was needed to control the infection and/or when death occurred due to the infection*”*.* Nearly all the patients who encountered side effects (43% of the total) needed either their antibiotic treatment changed or their dosage adjusted (Wouthuyzen-Bakker et al., 2017).

In our study, despite prolonged exposure to dalbavancin, adverse events were reported in only two patients (16 doses and 42 doses of DAL received), and in only one was dalbavancin decided to be discontinued. The rest of the patients, including those with a total load of 87, 62, and 48 DAL doses, referred no adverse events. Despite the effect of SAT on the microbiota not being evaluated, the incidence of adverse events (25%) was similar to the 32.8% and 20.1% reported by Boucher et al. (2014),; Dunne et al. (2016), respectively although higher than the incidence described by Wunsch et al. (2019) in their multicenter study of dalbavancin used for the treatment of prosthetic joint infection osteomyelitis and endocarditis. In this Austrian retrospective study on real-life dalbavancin use, authors reported adverse events in 3 out of 101 patients on dalbavancin. As in our cohort, only one patient after 11 weeks of treatment complained of severe fatigue, and in three patients a reversible increase in creatinine > 0.3 mg/dL from baseline was reported. Other adverse events as infusion-related reactions and persistent vertigo were also observed. The difference in the incidence of adverse events across studies could be affected by multiple factors such as the underlying conditions of the patients or the duration of treatment. For example, in the study reported by Wunsch et al., only four patients received 4, 8, 12, and 14 administrations of dalbavancin while the number of dalbavancin administrations was 1 or 2 in the rest of the patients. Also, the retrospective nature of these studies needs to be highlighted as the number of events relies on voluntary reporting by physicians and patients together with the fact that most adverse events were mild, and they may be under-reported.

The reduction in length of hospital stay is an obvious benefit of SAT when oral drugs are not an option due to resistance, drug-to-drug interactions, and poor vascular access, particularly in settings where OPAT programs are not available. In terms of cost, Bouza et al. (2018) estimated a potential cost-saving of €3,064 per Spanish patient by prescribing dalbavancin based on a 14-day antibiotic course and they concluded that this result is probably underestimated due to some direct and indirect costs not being included. By applying these broad cost analysis criteria, the median of estimated cost savings per patient due to the avoided hospital days seems to be beneficial, although, as previously mentioned, this is a descriptive cohort providing preliminary data and the cost of SAT with dalbavancin was not the aim of this report. For future accurate cost-effectiveness evaluations, more sophisticated analysis will be needed.

Our descriptive preliminary study has multiple limitations, as we describe very complex patients with different infections and managements in a retrospective manner. Also, there is potential selection bias of included patients. The lack of robust evidence relies on the fact that SAT is only used when no other surgical or therapy options are available for our patients. The development of randomized trials or large cohorts is desirable but complicated to execute in these scenarios without the support of large trial platforms and frequently, observational cohorts include a wide variety of complex patients. These issues impact the number of patients treated with SAT reported in the literature and evidence on prolonged use of antimicrobials is scarce. Frequently only case reports are available, and very few series are published. Some examples, regardless of the antimicrobial or the route of administration used, are the cohort reported by Beydoun et al. (2020), where the role of SAT in the management of spinal infections involving hardware was explored in 124 patients recruited over 10 years, although only 27 patients received antimicrobial > 12 months and 18 between 6 and 12 months; the retrospective cohort of 23 patients with prosthetic joint infections after hip replacement collated over 17 years by Leijtens et al. (2019); and the cohort published by Wouthuyzen-Bakker et al. (2017) with 21 patients with prosthetic joint infection (hip, knee or shoulder) on SAT, both in singles centers in the Netherlands. Where authors defined SAT as “any oral antibiotics given after completing the initial intravenous antibiotic regimen” and therefore, none of our patients could meet the criteria. Besides these cohorts, to date, publicly available data on long-term SAT experience is limited and based on small case reports. Some examples reporting the use of SAT are in “*a case of prosthetic valve endocarditis and a case of extensive aortic and subclavian graft infection*” (Lechner et al., 2020)*,* in an “*infected thoracic aorta graft*” (Berdal and Steinbakk, 2003)*,* in five patients with a proven or suspected infected abdominal aortic grafts (Roy and Grove, 2000) or in two “*rare cases of recurrent staphylococcal infections*” where azithromycin was used (Grobost et al., 2014). None of them included cost analysis. In our multicenter study, we finally managed to include eight patients with SAT indication, prosthesis infections, and receiving long-term dalbavancin.

The use of dalbavancin as SAT is even rarer and all co-authors were asked to provide all eligible cases available in their hospitals. The main purpose of this report is to share our experience with the prolonged use of this new agent, as no higher-quality evidence is available to date. To our knowledge, very limited experiences are reported with dalbavancin as a suppressive agent, mostly case reports (Spaziante et al., 2019; Barbero Allende et al., 2021; Matesanz et al., 2021; Pallotto et al., 2022) or smaller series like the one retrospectively described by Hitzenbichler et al. (2021) with four cases. Here, we report eight cases from different hospitals and with very prolonged therapies (as far as 21 months). Despite the limitations of our study, dalbavancin was observed to be an attractive candidate for SAT for patients with prosthetic infections in the outpatient setting in which the surgical treatment cannot be successfully performed and oral treatment is unavailable, and it supports further evaluation of dalbavancin for these scenarios.

## Data Availability

The original contributions presented in the study are included in the article/supplementary material, further inquiries can be directed to the corresponding author.
